# Supplementary material for: Content-rich biological network constructed by mining PubMed abstracts
Source: BMC Bioinformatics. 2004 Oct 8;5:147. doi: 10.1186/1471-2105-5-147 (PMC528731; doi:10.1186/1471-2105-5-147)
Supplement: Additional File 2 — The original results of the above study (non-essential files are deleted to keep the file size under the limit set by BMC bioinformatics). [file 1471-2105-5-147-S2.bz2 › chilibotAdditionalFile2/dip30/591ID9253415E10963/html/TUFA_TSF.html]

 


 **TUFA** and **TSF** 
  
Found 146 abstracts in PubMed, retrieved 30.  
 

 What does Google say? 
 PDF only 
| .edu only 

---

**Interactive relationship** (e.g. stimulation, inhibition, etc)

**Stimulatory relationship**- In reverse, phosphorylated EF Tu  [ **TUFA** ]  has an increased affinity for EF Ts  [ **TSF** ] , does not bind kirromycin and can no longer bind aminoacyi tRNA.  Ref: 10588048 Folia Microbiol Praha , 1999
- Recombinant nematode EF Ts  [ **TSF** ]  purified from Escherichia coli bound both of these molecules and also stimulated the translational activity of EF Tu  [ **TUFA** ] , indicating that the nematode EF Ts  [ **TSF** ]  homolog is a functional EF Ts  [ **TSF** ]  protein of mitochondria.  Ref: 12490713 Nucleic Acids Res, 2002
- EF Ts  [ **TSF** ]  enhances the dissociation of GDP and GTP from EF Tu  [ **TUFA** ]  by factors of 6 x 10 4 and 3 x 10 3, respectively.  Ref: 11772013 Biochemistry, 2002
- Elongation factor Ts  [ **TSF** ]  can act as a steric chaperone by increasing the solubility of nucleotide binding impaired elongation factor Tu  [ **TUFA** ] .  Ref: 11456491 Biochemistry, 2001
**Neutral relationship**- Characterization of the interaction between the nucleotide exchange factor EF Ts  [ **TSF** ]  from nematode mitochondria and elongation factor Tu  [ **TUFA** ] .  Ref: 12490713 Nucleic Acids Res, 2002
- This implies that stabilization of the EF Tu  [ **TUFA** ] .EF Ts  [ **TSF** ]  complex is related to the inability to bind Mg2, rather than to a decreased nucleotide affinity.  Ref: 10196198 J Biol Chem, 1999
- Roles of residues in mammalian mitochondrial elongation factor Ts  [ **TSF** ]  in the interaction with mitochondrial and bacterial elongation factor Tu  [ **TUFA** ] .  Ref: 9774433 J Biol Chem, 1998
- The EF Ts  [ **TSF** ]  bound to two eubacterial EF Tu  [ **TUFA** ]  species E.coli and Thermus thermophilus.  Ref: 12490713 Nucleic Acids Res, 2002
- Nucleotide exchange in elongation factor Tu  [ **TUFA** ]  EF Tu  [ **TUFA** ]  is catalyzed by elongation factor Ts  [ **TSF** ]  EF Ts  [ **TSF** ] .  Ref: 11744709 J Biol Chem, 2002
- This indicates that nematode EF Ts  [ **TSF** ]  can be used to stabilize homologous EF Tu  [ **TUFA** ]  molecules for experimental purposes.  Ref: 12490713 Nucleic Acids Res, 2002
- Elongation factor Ts  [ **TSF** ]  EF Ts  [ **TSF** ]  will catalyze the exchange of nucleotide on EF Tu  [ **TUFA** ] .  Ref: 10397336 Acta Biochim Pol, 1998
- Interaction of mitochondrial elongation factor Tu  [ **TUFA** ]  with aminoacyl tRNA and elongation factor Ts  [ **TSF** ] .  Ref: 10801827 J Biol Chem, 2000
- For example, F19A I20A and H176A derivatives of EF Tsmt are as active as E. coli EF Ts  [ **TSF** ]  in simulating E. coli EF Tu  [ **TUFA** ] .  Ref: 9774433 J Biol Chem, 1998
- In contrast to earlier experiments, purification of free mitochondrial EF Ts  [ **TSF** ]  was accomplished under nondenaturing conditions since only about 60% of the expressed EF Ts  [ **TSF** ]  copurified with E. coli EF Tu  [ **TUFA** ] .  Ref: 12445479 Biochim Biophys Acta, 2002
- Evidence that a single EF Ts  [ **TSF** ]  suffices for the recycling of multiple and divergent EF Tu  [ **TUFA** ]  species in Streptomyces coelicolor A3 2 and Streptomyces ramocissimus.  Ref: 10517582 Microbiology, 1999
- Some of them are in the regions of interdomain interactions, others correlate with the known functionally important regions of EF Tu  [ **TUFA** ]  binding with EF Ts  [ **TSF** ] , aminoacyl tRNA and the ribosome.  Ref: 11771276 Biofizika< MedlineTA>Biofizika, 1999
- Elongation factor EF Tu  [ **TUFA** ]  alternates between two interaction partners, EF Ts  [ **TSF** ]  and the ribosome, during its functional cycle.  Ref: 11231892 J Mol Evol, 2001
- Elongation factor Ts  [ **TSF** ]  EF Ts  [ **TSF** ]  promotes the formation of active GTP bound elongation factor Tu  [ **TUFA** ]  EF Tu  [ **TUFA** ]  by accelerating the dissociation of GDP from the EF Tu  [ **TUFA** ]  x GDP complex.  Ref: 9692904 Eur J Biochem, 1998
- Thus, the nematode EF Ts  [ **TSF** ]  appears to have a broad specificity for EF Tu  [ **TUFA** ]  molecules from different species.  Ref: 12490713 Nucleic Acids Res, 2002
- Of the mutants, only EF Tu  [ **TUFA** ]  T25A forms a more stable complex with EF Ts  [ **TSF** ]  than wild type.  Ref: 10196198 J Biol Chem, 1999
- 50 656 in which each EF Tu  [ **TUFA** ]  in the dyad symmetrical heterotetrameric complex interacts with two subunits of EF Ts  [ **TSF** ] .  Ref: 9692904 Eur J Biochem, 1998
- Dimers of Thermus thermophilus elongation factor Ts  [ **TSF** ]  are required for its function as a nucleotide exchange factor of elongation factor Tu  [ **TUFA** ] .  Ref: 9692904 Eur J Biochem, 1998
- A common structural motif in elongation factor Ts  [ **TSF** ]  and ribosomal protein L7 12 may be involved in the interaction with elongation factor Tu  [ **TUFA** ] .  Ref: 11231892 J Mol Evol, 2001
- The kinetic results suggest that the initial contact of EF Ts  [ **TSF** ]  with helix D of EF Tu  [ **TUFA** ]  weakens binding interactions around the guanine base.  Ref: 11744709 J Biol Chem, 2002
- The structure of the bacterial analog of EF 1alpha, EF Tu  [ **TUFA** ]  has been solved in complex with its GDP exchange factor, EF Ts  [ **TSF** ] .  Ref: 10368288 Structure Fold Des, 1999
- In contrast, the presence of any of the three domains of E. coli EF Tu  [ **TUFA** ]  in EF Tumt allowed the mitochondrial factor to interact with bacterial EF Ts  [ **TSF** ] .  Ref: 10395923 Biochim Biophys Acta, 1999
- EF Ts  [ **TSF** ]  variants which were unable to form dimers were also inactive in the nucleotide exchange on EF Tu  [ **TUFA** ] .  Ref: 9692904 Eur J Biochem, 1998
- During the elongation cycle, EF Tu  [ **TUFA** ]  interacts with guanine nucleotides, aa tRNA and its nucleotide exchange factor EF Ts  [ **TSF** ] .  Ref: 11045624 Protein Sci, 2000
- Rate constants of EF Ts  [ **TSF** ]  binding to EF Tu  [ **TUFA** ]  in the free or nucleotide bound form or of GDP GTP binding to the EF Tu  [ **TUFA** ] .EF Ts  [ **TSF** ]  complex range from 0.6 x 10 7 to 6 x 10 7 M 1 s 1.  Ref: 11772013 Biochemistry, 2002
- We present here results showing that the co overexpression of EF Ts  [ **TSF** ]  at a 1 1 ratio dramatically improves the solubility of mutant EF Tu  [ **TUFA** ] , although in the case of EF Tu  [ **TUFA** ]  D138N which cannot at all bind the nucleotides available in the cell this is a slow process.  Ref: 11456491 Biochemistry, 2001
- The crystal structure of the complex between Escherichia coli elongation factors Tu and Ts EF Tu  [ **TUFA** ] .Ts and subsequent mutagenesis work have provided insights into the roles of a number of residues in E. coli EF Ts  [ **TSF** ]  in its interaction with EF Tu  [ **TUFA** ] .  Ref: 9774433 J Biol Chem, 1998
- Dissociation of EF Ts  [ **TSF** ]  from the ternary complexes with EF Tu  [ **TUFA** ]  and GDP GTP is 10 3 10 4 times faster than from the binary complex EF Tu  [ **TUFA** ] .EF Ts  [ **TSF** ] , indicating different structures and or interactions of the factors in the binary and ternary complexes.  Ref: 11772013 Biochemistry, 2002
- Most amino acid residues known from the Escherichia coli crystal structure of the EF Tu  [ **TUFA** ]  EF Ts  [ **TSF** ]  complex to be directly involved in interaction between the two elongation factors are conserved between E. coli and Streptomyces.  Ref: 10517582 Microbiology, 1999
- Using this EF Tu  [ **TUFA** ]  dependent translation system, we have established that S. coelicolor EF Tu3 has translational capacities despite its striking deviation from the common prokaryotic EF Tu  [ **TUFA** ]  sequence at positions involved in either aminoacyl tRNA binding or interaction with the guanine nucleotide exchange factor EF Ts  [ **TSF** ] .  Ref: 11432749 Eur J Biochem, 2001
- Escherichia coli elongation factor EF Tu  [ **TUFA** ]  and the corresponding mammalian mitochondrial factor, EF Tumt, show distinct differences in their affinities for guanine nucleotides and in their interactions with elongation factor Ts  [ **TSF** ]  EF Ts  [ **TSF** ]  and mitochondrial tRNAs.  Ref: 10395923 Biochim Biophys Acta, 1999
- Moreover, EF Tu  [ **TUFA** ]  T25A and EF Tu  [ **TUFA** ]  D80N were shown to inhibit cell growth on expression, an effect attributed to their sequestration of EF Ts  [ **TSF** ]  Krab, I.  Ref: 11456491 Biochemistry, 2001

**Non-interactive relationship** (e.g. studied together, co-existance, homology, etc.)

- K Ts was determined by quantifying the effects of increasing concentrations of EF Ts  [ **TSF** ]  mt on the amount of ternary complex formed with EF Tu  [ **TUFA** ]  mt.  Ref: 10801827 J Biol Chem, 2000
- Previous studies have shown that when bovine mitochondrial elongation factor Ts  [ **TSF** ]  EF Ts  [ **TSF** ]  is expressed in Escherichia coli, it forms a tightly associated complex with E. coli elongation factor Tu  [ **TUFA** ]  EF Tu  [ **TUFA** ] .  Ref: 12445479 Biochim Biophys Acta, 2002
- Mechanism of elongation factor EF Ts  [ **TSF** ]  catalyzed nucleotide exchange in EF Tu  [ **TUFA** ] .  Ref: 11744709 J Biol Chem, 2002
- The interaction of Escherichia coli elongation factor Tu  [ **TUFA** ]  EF Tu  [ **TUFA** ]  with elongation factor Ts  [ **TSF** ]  EF Ts  [ **TSF** ]  and guanine nucleotides was studied by the stopped flow technique, monitoring the fluorescence of tryptophan 184 in EF Tu  [ **TUFA** ]  or of the mant group attached to the guanine nucleotide.  Ref: 11772013 Biochemistry, 2002
- Although the nematode mitochondrial mt EF Tu  [ **TUFA** ]  molecules are extremely unstable and easily form aggregates, native gel electrophoresis and gel filtration analysis revealed that EF Tu  [ **TUFA** ] .EF Ts  [ **TSF** ]  complexes are significantly more soluble.  Ref: 12490713 Nucleic Acids Res, 2002
- The crystals of the homologous mitochondrial EF Tu  [ **TUFA** ]  EF Ts  [ **TSF** ]  complex diffract to 4 A and belong to space group C2 with cell parameters a=157.7 A, b=151.9 A, c=156.9 A, and beta=108.96 degrees.  Ref: 12445479 Biochim Biophys Acta, 2002
- We prepared antibodies against EF Tu  [ **TUFA** ]  mt and EF Ts  [ **TSF** ]  mt using purified recombinant EF Tu  [ **TUFA** ]  mt and EF Ts  [ **TSF** ]  mt.  Ref: 12142912 Sheng Wu Hua Xue Yu Sheng Wu Wu Li Xue Bao Shanghai < MedlineTA>Sheng Wu Hua Xue Yu Sheng Wu Wu Li Xue Bao Shanghai , 2002
- In the present paper, we determine the contribution of the contacts between helix D of EF Tu  [ **TUFA** ]  at the base side of the nucleotide and the N terminal domain of EF Ts  [ **TSF** ]  to the catalysis.  Ref: 11744709 J Biol Chem, 2002
- The biochemical data presented here are supported by the recently solved structure of the T. thermophilus EF Tu  [ **TUFA** ]  x EF Ts  [ **TSF** ]  complex Wang, Y.  Ref: 9692904 Eur J Biochem, 1998
- Isolation, crystallisation, and preliminary X ray analysis of the bovine mitochondrial EF Tu  [ **TUFA** ]  GDP and EF Tu  [ **TUFA** ]  EF Ts  [ **TSF** ]  complexes.  Ref: 12445479 Biochim Biophys Acta, 2002
- Exchange of GDP for GTP is carried out through the formation of a complex with EF Ts  [ **TSF** ]  EF Tu  [ **TUFA** ] .Ts.  Ref: 10801827 J Biol Chem, 2000
- The loss of Mg 2 alone, without EF Ts  [ **TSF** ] , accounts for a 150 300 fold acceleration of GDP dissociation from EF Tu  [ **TUFA** ] .GDP, suggesting that the disruption of the Mg 2 binding site alone does not explain the EF Ts  [ **TSF** ]  effect.  Ref: 11772013 Biochemistry, 2002
- Streptomycetes have multiple and highly divergent EF Tu  [ **TUFA** ]  species, with EF Tu1 and EF Tu3 showing only about 65% amino acid sequence identity, and yet these can apparently interact with a single EF Ts  [ **TSF** ]  species.  Ref: 10517582 Microbiology, 1999
- Their replacement by Asp, Ala and Asp, respectively, influences to different degrees the stability of EF Ts  [ **TSF** ] , the ability of EF Ts  [ **TSF** ]  to form dimers, and the ability to interact with EF Tu  [ **TUFA** ] .  Ref: 9692904 Eur J Biochem, 1998
- Several proteins, including the elongation factors EF G,  **TufA**  and EF Ts  [ **TSF** ] , were found to be highly carbonylated.  Ref: 12845527 Mol Genet Genomics< MedlineTA>Mol Genet Genomics, 1998
- The bovine mitochondrial EF Tu  [ **TUFA** ]  GDP complex, the homologous mitochondrial EF Tu  [ **TUFA** ]  EF Ts  [ **TSF** ]  complex, and the heterologous E. coli mitochondrial EF Tu  [ **TUFA** ]  EF Ts  [ **TSF** ]  complex were isolated and crystallised.  Ref: 12445479 Biochim Biophys Acta, 2002
- Mutational analysis shows that contacts between helix D of EF Tu  [ **TUFA** ]  and the N terminal domain of EF Ts  [ **TSF** ]  are important for both complex formation and the acceleration of GDP dissociation.  Ref: 11744709 J Biol Chem, 2002
- We conclude that for the formation of a correct EF Tu  [ **TUFA** ]  structure the nucleotide plays an important role as a folding nucleus, and also that in its absence EF Ts  [ **TSF** ]  can act as a folding template or steric chaperone for the correct folding of EF Tu  [ **TUFA** ] .  Ref: 11456491 Biochemistry, 2001
- Although the EF Ts  [ **TSF** ]  did not bind to bovine mt EF Tu  [ **TUFA** ] , it could bind to a chimeric nematode bovine EF Tu  [ **TUFA** ]  molecule containing domains 1 and 2 from bovine mt EF Tu  [ **TUFA** ] .  Ref: 12490713 Nucleic Acids Res, 2002
- The activity of E. coli EF Tu  [ **TUFA** ]  but not of EF Tumt is stimulated by E. coli EF Ts  [ **TSF** ] .  Ref: 10395923 Biochim Biophys Acta, 1999
- We demonstrate that the transfer of fully charged aminoacyl tRNAs into peptides directed by the MS2 RNA template requires both ATP and GTP, initiation factors IF1, IF2, and IF3, elongation factors EF Tu  [ **TUFA** ] , EF Ts  [ **TSF** ] , and EF G, and the ribosomal ATPase RbbA.  Ref: 11557474 Antimicrob Agents Chemother, 2001
- However, whenever interaction residues in the EF Tu  [ **TUFA** ]  moiety show divergence among Streptomyces EF Tu1, EF Tu2 and EF Tu3, the single Streptomyces EF Ts  [ **TSF** ]  exhibits compensatory substitutions of the corresponding residues.  Ref: 10517582 Microbiology, 1999
- The rate constants of the multistep reaction between Escherichia coli EF Tu  [ **TUFA** ] , EF Ts  [ **TSF** ] , and GDP were determined by stopped flow kinetic analysis monitoring the fluorescence of either Trp 184 in EF Tu  [ **TUFA** ]  or mant GDP.  Ref: 11744709 J Biol Chem, 2002
- Rate constants of all association and dissociation reactions among EF Tu  [ **TUFA** ] , EF Ts  [ **TSF** ] , GDP, and GTP were determined.  Ref: 11772013 Biochemistry, 2002
- Identification of two proteins differentially expressed by C. burnetii, EF Tu  [ **TUFA** ]  and EF Ts  [ **TSF** ] , both essential components of the translational machinery of the cell, supports the hypothesis that LCVs are metabolically more active than SCVs.  Ref: 10531263 Infect Immun, 1999
- Kinetic mechanism of elongation factor Ts  [ **TSF** ]  catalyzed nucleotide exchange in elongation factor Tu  [ **TUFA** ] .  Ref: 11772013 Biochemistry, 2002
- The mitochondrial elongation factors Tu and Ts EF Tu  [ **TUFA** ]  mt and EF Ts  [ **TSF** ]  mt are nuclear gene encoded mitochondrial proteins and play important roles in growth and development.  Ref: 12142912 Sheng Wu Hua Xue Yu Sheng Wu Wu Li Xue Bao Shanghai < MedlineTA>Sheng Wu Hua Xue Yu Sheng Wu Wu Li Xue Bao Shanghai , 2002
- The binding of EF Tu  [ **TUFA** ]  mt to EF Ts  [ **TSF** ]  mt is mutually exclusive with the formation of the ternary complex.  Ref: 10801827 J Biol Chem, 2000
- Microcalorimetric study of elongation factor Tu  [ **TUFA** ]  from Thermus thermophilus in nucleotide free, GDP and GTP forms and in the presence of elongation factor Ts  [ **TSF** ] .  Ref: 12007615 Biochim Biophys Acta, 2002
- The relative abundance of GFM was consistent with the observations for human EF Tu  [ **TUFA** ]  mt and EF Ts  [ **TSF** ]  mt, the other two mitochondrial elongation factors, indicating that the three factors were expressed at corresponding levels.  Ref: 11374907 Genomics, 2001
- Binding of EF Tu  [ **TUFA** ] .GDP to aminoacyl tRNA and mutually exclusive binding of kirromycin and elongation factor Ts  [ **TSF** ]  to EF Tu  [ **TUFA** ]  can be explained on the basis of the structure.  Ref: 11278992 J Biol Chem, 2001
- These results suggest that expression levels of EF Tu  [ **TUFA** ]  mt and EF Ts  [ **TSF** ]  mt may be regulated by a similar mechanism related to the metabolic state of the cells.  Ref: 12142912 Sheng Wu Hua Xue Yu Sheng Wu Wu Li Xue Bao Shanghai < MedlineTA>Sheng Wu Hua Xue Yu Sheng Wu Wu Li Xue Bao Shanghai , 2001
- Although there is good sequence conservation between EF 1alpha and EF Tu  [ **TUFA** ] , there is essentially no sequence similarity between EF 1beta and EF Ts  [ **TSF** ] .  Ref: 10368288 Structure Fold Des, 1999
- Certain proteins taking part in the translation process, such as the elongation factors EF Tu  [ **TUFA** ] , EF Ts  [ **TSF** ]  and the ribosomal protein RP L9, were identified by Edman N terminal sequencing and have thus been considered for reaction evaluation.  Ref: 10344251 Electrophoresis< MedlineTA>Electrophoresis, 1999
- Expression of EF Tu  [ **TUFA** ]  mt and EF Ts  [ **TSF** ]  mt exhi bited similar tissue specificity and changed synchronically during development.  Ref: 12142912 Sheng Wu Hua Xue Yu Sheng Wu Wu Li Xue Bao Shanghai < MedlineTA>Sheng Wu Hua Xue Yu Sheng Wu Wu Li Xue Bao Shanghai , 1999
- Western analysis indicates EF Tu  [ **TUFA** ]  mt and EF Ts  [ **TSF** ]  mt are expressed differently in various tissues.  Ref: 12142912 Sheng Wu Hua Xue Yu Sheng Wu Wu Li Xue Bao Shanghai < MedlineTA>Sheng Wu Hua Xue Yu Sheng Wu Wu Li Xue Bao Shanghai , 1999
- The proteins containing such sequence signatures include 1 the DnaJ Hsp40 family of proteins, 2 DNA polymerase I, 3 the protein synthesis elongation factor EF Tu  [ **TUFA** ] , and 4 the elongation factor EF Ts  [ **TSF** ] .  Ref: 9608054 J Mol Evol, 1998
